# Long non‐coding RNA deleted in lymphocytic leukaemia 1 promotes hepatocellular carcinoma progression by sponging miR‐133a to regulate IGF‐1R expression

**DOI:** 10.1111/jcmm.14384

**Published:** 2019-06-17

**Authors:** Wei Zhang, Songyang Liu, Kai Liu, Yahui Liu

**Affiliations:** ^1^ Department of Hepatopancreatobiliary Surgery The First Hospital of Jilin University Changchun P.R. China

**Keywords:** DLEU1, hepatocellular carcinoma, IGF‐1R, LncRNAs, miR‐133a, PI3K/AKT

## Abstract

Long non‐coding RNA (lncRNA) deleted in lymphocytic leukaemia 1 (DLEU1) was reported to be involved in the occurrence and development of multiple cancers. However, the exact expression, biological function and underlying mechanism of DLEU1 in hepatocellular carcinoma (HCC) remain unclear. In this study, real‐time quantitative polymerase chain reaction (qRT‐PCR) in HCC tissues and cell lines revealed that DLEU1 expression was up‐regulated, and the increased DLEU1 was closely associated with advanced tumour‐node‐metastasis stage, vascular metastasis and poor overall survival. Function experiments showed that knockdown of DLEU1 significantly inhibited HCC cell proliferation, colony formation, migration and invasion, and suppressed epithelial to mesenchymal transition (EMT) process via increasing the expression of E‐cadherin and decreasing the expression of N‐cadherin and Vimentin. Luciferase reporter gene assay and RNA immunoprecipitation (RIP) assay demonstrated that DLEU1 could sponge miR‐133a. Moreover, miR‐133a inhibition significantly reversed the suppression effects of DLEU1 knockdown on HCC cells. Besides, we found that silenced DLEU1 significantly decreased insulin‐like growth factor 1 receptor (IGF‐1R) expression (a target of miR‐133a) and its downstream signal PI3K/AKT pathway in HCC cells, while miR‐133a inhibitor partially reversed this trend. Furthermore, DLEU1 knockdown impaired tumour growth in vivo by regulating miR‐133a/IGF‐1R axis. Collectively, these findings indicate that DLEU1 promoted HCC progression by sponging miR‐133a to regulate IGF‐1R expression. Deleted in lymphocytic leukaemia 1/miR‐133a/IGF‐1R axis may be a novel target for treatment of HCC.

## 1 INTRODUCTION

Hepatocellular carcinoma (HCC) is the fifth most common malignant tumour and the second leading cause of cancer‐related death worldwide, with high morbidity and high mortality.^1^ Despite great advances in the surgical treatment and chemo‐radiotherapy, the outcome of HCC patients remain unsatisfactory mainly due to recurrence and distal metastasis after treatments.^2^, ^3^ Therefore, it is imperative to elucidate the key molecular mechanisms of the pathogenesis and development of HCC for finding new therapeutic strategies for this disease.

Long non‐coding RNAs (lncRNAs), a prominent class of transcripts longer than 200 nucleotides in length and limited protein‐coding potential,^4^ have recently gained wide attention due to their functional roles in a variety of biological processes.^5^, ^6^ Long non‐coding RNAs have been highlighted to be involved in the occurrence and development of cancer,^7^, ^8^ offering the possibility of lncRNAs as novel diagnosis markers and therapy agent for cancer. Number of lncRNAs was identified to function as oncogene or tumour suppressors in HCC by modulation of cell proliferation, autophagy, apoptosis, cycle, invasion and metastasis via different pathway.^9^, ^10^

Long non‐coding RNA deleted in lymphocytic leukaemia 1 (DLEU1), located on chromosome 13q14.3,^11^ has been reported to be up‐regulated and function as oncogene in several types of cancer, including oral squamous cell carcinoma,^12^ colorectal cancer,^13^ gastric cancer,^14^ ovarian cancer^15^ and endometrial carcinoma.^16^ However, the role and potential molecular mechanism of DLEU1 in HCC remain unclear. Therefore, the aims of this study were to investigate the role of DLEU1 in HCC progression and explore the mechanism behind it in HCC action.

## 2 MATERIALS AND METHODS

### 2.1 Tissue specimens

A total of 56 HCC tissues and paired adjacent tissues were collected from patients with primary HCC with no previous others treatment who underwent curative resection between January 2013 and August 2014 at the Department of Hepatopancreatobiliary Surgery, the First Hospital of Jilin University. All tissues were rapidly frozen in liquid nitrogen following surgery and stored at the temperature of −80°C until RNA extraction. Tumour pathology and staging were determined by two independent pathologists form our hospital. Prior to operation, none of the patients received chemo‐ or radiotherapy and other therapy. The Clinical Research Ethics Committee of Jilin University approved this study. Informed consent was signed by each patient enroled in this study. Table 1 summarizes the relevant clinicopathological characteristics of all patients with HCC.

**Table 1:** Association of DLEU1 expression with clinicopathologic factors of 56 patients with HCC

Variables	No. of cases	DLEU1 expression		*P* value
		High (n %)	Low (n %)	
Age (y)				0.5813
<50	22	14 (63.6)	8 (36.4)	
≥50	34	18 (52.9)	16 (47.1)	
Gender				0.7889
Male	31	17 (54.8)	14 (45.2)	
Female	25	15 (60.0)	10 (40.0)	
TNM stage				<0.0001
I‐II	42	18 (42.8)	24 (52.4)	
III‐IV	14	14 (100.0)	0 (0)	
Differentiated				0.2504
Well/moderate	38	24 (63.2)	14 (37.8)	
Poor	18	8 (44.4)	10 (55.6)	
Serum AFP (ng/mL)				0.4184
≤20	26	13 (50.0)	13 (50.0)	
>20	30	19 (63.3)	11 (36.7)	
HCV antigen				0.1778
Positive	34	22 (64.7)	12 (35.3)	
Negative	22	10 (45.4)	12 (54.6)	
Vascular invasion				0.0161
No	45	22 (48.9)	23 (52.1)	
Yes	11	10 (90.9)	1 (9.1)	

Abbreviations: AFP, α‐fetoprotein; DLEU1, deleted in lymphocytic leukaemia 1; HCC, hepatocellular carcinoma; HCV, hepatitis C virus; TNM, tumour‐node‐metastasis.

### 2.2 Cell culture and transfection

Institute of Cell Biology of Chinese Academy of Science (Shanghai, China) was the source of human HCC cell lines (SMMC‐7721, Hep3B, HepG2, Huh‐7) and normal hepatic epithelial cell line LO2. Cells were grown in a humidified 5% CO_2_ incubator in DMEM (Gibco, USA) containing 10% heat‐inactivated fetal bovine serum (FBS) (HyClone, Logan, UT), and penicillin/streptomycin (100 U/mL; Sigma‐Aldrich, St. Louis, MO) at 37°C.

Short hairpin RNA (shRNA) directed against DLEU1 (sh‐DLEU1) and scrambled shRNA control (sh‐NC) were designed and inserted into the GV248 vector by Genechem (Shanghai, China), then were transfected into SMMC‐7721 and HepG2 cells using lipofectamine 3000 transfection reagent (Invitrogen, Carlsbad, CA) following the manufacturer's protocol. Stable clones with sh‐DLEU1 or sh‐NC in SMMC‐7721 and HepG2 cells were selected for 4 weeks using 800 μg/mL neomycin (Sigma‐Aldrich). miR‐133a mimic (miR‐133a), scrambled miRNA negative control (miR‐NC), miR‐133a inhibitor (anti‐miR‐133a) and scrambled inhibitor control (anti‐miR‐NC) were purchased from GenePharma Co., Ltd. (Shanghai, China), and were also underwent transfection into cells as above.

### 2.3 RNA extraction and quantitative real‐time polymerase chain reaction

Total RNA was purified from clinical samples and cultured cell lines based on the protocol of TRIzol reagent (Invitrogen). For detection of miR‐133a expression, cDNA was synthesized using One Step Prime script miRNA cDNA Synthesis Kit (Qiagen, Hilden, Germany) following the manufacturer's instructions. Quantitative polymerase chain reaction (qPCR) was conducted using the miScript SYBR Green PCR Kit (Qiagen) under the ABI 7900 qPCR System (Applied Biosystems, Foster, CA). For detection of mRNA expression, cDNAs were synthesized using a reverse transcription kit (Takara, Dalian, China). Quantitative polymerase chain reaction was performed by the SYBR Green Real‐Time PCR Master Mix (Roche, Basel, Switzerland) the manufacturer's instructions. All primes used in this study are listed in Table 2. Glyceraldehyde‐3‐phosphate dehydrogenase (GAPDH) and U6 small nuclear RNA were used as internal controls. Relative expression levels were calculated based on 2^−∆∆Ct^ method from three independent experiments.

**Table 2:** Real time PCR primers used for mRNA expression analysis

Target gene	Prime(5′‐3′)
U6	F‐TCCGATCGTGAAGCGTTC R‐GTGCAGGGTCCGAGGT
miR‐133a	F‐GTGCTTTGGTCCCCTTCAAC R‐GCAGGGTCCGAGGTATTC
DLEU1	F‐CACGTGCATTTAAAACCGCC R‐AAGACTTTGGGGCAGATTTCTT
IGF‐1R	F‐TTTCCCACAGCAGTCCACCTC R‐AGCATCCTAGCCTTCTCACCC
GAPDH	F‐AAGGTGAAGGTCGGAGTCAA R‐AATGAAGGGGTCATTGATGG

Abbreviations: DLEU1, deleted in lymphocytic leukaemia 1; F, forward; GAPDH, glyceraldehyde‐3‐phosphate dehydrogenase; IGF‐1R, insulin‐like growth factor 1 receptor; mRNA, messenger RNA; PCR, polymerase chain reaction; R, reverse.

### 2.4 Cell Counting kit‐8 assay

Cell proliferation was determined by Cell Counting kit‐8 (CCK8) assay. Briefly, SMMC‐7721 or HepG2 cells (5 × 10^3^ cells/well) were seeded into 24‐well plates, then were incubated in 10% CCK8 solution (Dojindo, Kumamoto, Japan) at 72 hours after transfection, which were incubated at 37°C for another 2‐4 hours until visual colour conversion occurred. A microplate spectrophotometer (BioTek Instruments, Winooski, VT) was then used to assess absorbance at 450 nm.

### 2.5 Colony formation assay

Stable DLEU1‐depleted SMMC‐7721/HepG2 cells were incubated in six‐well plates at density of 1000 cells/well, and maintained in DMEM medium containing 10% FBS, replacing the medium every 3 days. After 14 days, the colonies were fixed with 96% ethanol and stained with 1% crystal violet for 5 minutes. The visible colonies were manually imaged and counted using a light microscope X71 (Olympus Corporation, Tokyo, Japan).

### 2.6 Flow cytometric analysis of cell cycle distribution

Transfected cells were fixed with 70% ethanol and incubated with staining solution containing RNase A (200 μg/mL) and propidium iodide (PI; 50 μg/mL; Sigma‐Aldrich) for 15 minutes at 4°C. A FACSCalibur flow cytometer (Beckman Coulter, Fullerton, CA) was used to determine cell arrest distribution.

### 2.7 Wound healing assay

After seeding (5 × 10^5^ cells/well, six‐well plate), wounding was achieved by scratching with a sterile 200 μL pipette tip. Serum‐free medium was then added, and cells were allowed to move towards the denuded area for 24 hours. The spread of the wound was observed under light microscopy and photographed at 0 and 24 hours. The wound areas were analysed using Imagej software 3.2 (National Institutes of Health, Bethesda, MD).

### 2.8 Transwell invasion assay

Matrigel‐coated Transwell cell culture chambers (BD Bioscience, San Jose, CA) were used for the invasion assay. Briefly, transfected cells (5 × 10^5^ cells/well) re‐suspended in serum‐free medium were placed into in the upper compartment of the Transwell inserts. DMEM medium containing 20% FBS was added to the lower chambers serving as the chemoattractant. After 48 hours of incubation at 37°C, the invaded cells were fixed with formaldehyde, and stained with 1% crystal violet. Stained cells were observed and photographed under Olympus fluorescence microscope (Tokyo, Japan), counted and calculated the mean in five randomly selected fields.

### 2.9 Luciferase reporter assay

Starbase 2.0 (http://starbase.sysu.edu.cn/) was used to analyse the miR‐133a binding sites on DLEU1. Luciferase assay was performed to validate prediction of miR‐133a as a target of DLEU1. The wide‐type 3′ untranslated region (UTR) of DLEU1 and the mutant 3′UTR of DLEU1 were synthesized and cloned into the downstream of pGL3/Luciferase vector (Ambion, Austin, TX), and named as: Wt‐DLEU1 and Mut‐DLEU1 respectively. For luciferase reporter assay, SMMC‐7721 or HepG2 cells were cultured in 96‐well plates until 80% confluence. Plasmid vector including Wt‐DLEU1 or Mut‐DLEU1 together with miR‐133a mimics or miR‐NC were co‐transfected into SMMC‐7721 cells using the Lipofectamine 3000 reagent. Dual Luciferase Assay Kit (Promega, Madison, WI) was employed to examine luciferase activity after transfection for 48 hours. Renilla‐luciferase activity was used as control.

### 2.10 RNA immunoprecipitation

RNA immunoprecipitation (RIP) experiments was performed with the Magna RIP™RNA‐Binding Protein Immunoprecipitation Kit (Millipore, USA) based on the manufacturer's instructions. Briefly, the cells were lysed in a RIP lysis buffer for 30 minutes. Then cell lysate were incubated with RIP buffer containing magnetic beads conjugated with Ago2 antibody (Abcam, Cambridge, MA) and NC normal mouse IgG (Abcam). The co‐precipitated RNAs were determined by qRT‐PCR. The total RNAs were used as the input controls.

### 2.11 Western blot

Total proteins were extracted from cultured cells and tumour tissues by lysis buffer (Beyotime, Beijing, China) base on the manufacturer's instructions. Concentrations of protein were measured using a BCA assay kit (Pierce, Rockford, IL). Protein extracts (30 μg per lane) were separated on 8%‐12% SDS‐polyacrylamide gels and then transferred onto polyvinylidene difluoride membrane (Bio‐Rad, Hercules, CA). After a 2‐hour blocking step using 5% skim milk, blots were probed using following primary antibodies overnight at 4°C: anti‐insulin‐like growth factor 1 receptor (IGF‐1R) (Santa Cruz, USA), anti‐phospho‐Akt (p‐Akt) (Cell Signaling), anti‐Akt (Cell Signaling), anti‐phospho‐PI3K (p‐PI3K) (Cell Signaling), anti‐PI3K (Cell Signaling), anti‐E‐cadherin (1:1000; Santa Cruz), anti‐N‐cadherin (Santa Cruz), anti‐Vimentin (Santa Cruz) and anti‐GAPDH (Santa Cruz). GAPDH was used as the control. After incubation with corresponding secondary antibody horseradish peroxidase (HRP) conjugation, the protein signals were observed using an enhanced chemiluminescence‐based FluorChem^®^ FC2 imaging system (Alpha Innotech, San Jose, CA). Relative protein expression levels were analysed using the Imagej software (National Institutes of Health).

### 2.12 Tumour formation in BALB/c nude mice

Male athymic 5‐week‐old BALB/c nude mice (18‐20 g) were bought from the Experiments Animal Center of Changchun Biological Institute (Changchun, China) and were kept in specific pathogen‐free conditions. All experiments for the in vivo nude mouse study were performed in accordance with the Guide for the Care and Use of Laboratory Animals of the National Institute of Health and were approved by the Animal Ethics Committee of Jilin University (Changchun, China).

Ten mice were randomly divided into two groups (each group five mice), and received 2 × 10^6^ SMMC‐7721 cells that had undergone stable transfection using sh‐SHNG3 or sh‐NC via subcutaneously injected respectively. Tumour volume was measured every 7 days by the formula: 0.5 × length × width^2^. Thirty‐five days after injection, mice were killed, and the tumours were removed, photographed and partially the xenograft tumour processed for qRT‐PCR and western blotted, as well as partially fixed for immunohistochemical staining.

### 2.13 Immunohistochemistry

Immunostaining was performed on the paraffin‐embedded tumour tissues from nude mice using streptavidin‐peroxidase conjugated method as described previously.^17^ Ki67 antibody was purchased from Abcam (Cambridge, UK).The sections were visualized under the light microscope (Olympus) at 200×.

### 2.14 Statistical analysis

All results are showed as mean ± SD from at least three replicates measurements, and were analysed using spss v17.0 (IBM, Chicago, IL) and Graphpad Prism6.0 (San Diego, CA). Data from two groups were compared with Student's *t* tests, while one‐way ANOVAs with Bonferroni's correction were used for comparisons between three or more groups. Kaplan‐Meier method and log‐rank test were applied to analysis overall survival ratio. Pearson's correlation coefficient was used to analyse correlation between two groups. The threshold of significance was *P < 0.05, **P < 0.01.

## 3 RESULTS

### 3.1 DLEU1 was up‐regulated in HCC tissues and was associated with the poor survival of patients

**Figure 1:**
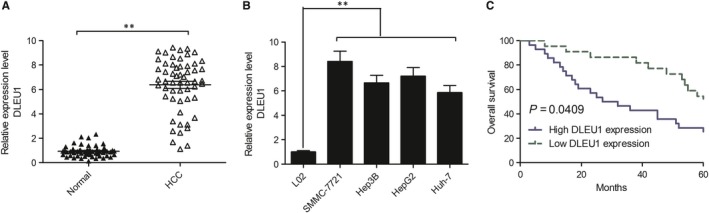
Deleted in lymphocytic leukaemia 1 (DLEU1) was up‐regulated in hepatocellular carcinoma (HCC) tissues and was associated with the poor survival of patients. A, Relative expression of DLEU1 was up‐regulated in human HCC cell lines (SMMC‐7721, Hep3B, HepG2, Huh‐7) compared to normal hepatic epithelial cell line LO2. B, Relative expression of DLEU1 was up‐regulated in HCC tissues compared to adjacent normal tissues. C, Kaplan‐Meier curves of the overall survival of HCC patients with high and low expression of DLEU1. **P < 0.01

We examined the DLEU1 expression in HCC cell lines to analyse DLEU1 expression in hepatic carcinogenesis and progression. Results of qRT‐PCR revealed that DLEU1 expressions were remarkably increased in HCC cell lines relative to LO2 cells lines (Figure 1A). Moreover, the expression levels of DLEU1 were remarkably increased in HCC tissues compared with adjacent normal tissues (Figure 1B). To further explore the clinical significance of DLEU1 expression in HCC, we divided all the patients into two groups based on median of DLEU1 expression: high DLEU1 expression group (>median DLEU1 expression, n = 32) and low DLEU1 expression group (<median DLEU1 expression, n = 24). The association between DLEU1 expression and clinical features is analysed and summarized in Table 1. There was no significant association between DLEU1 expression and patient's age, gender, differentiated, serum α‐fetoprotein (AFP) and hepatitis C virus (HCV) antigen (All *P* > 0.05, Table1). However, increased DLEU1 was positively associated with vascular invasion and tumour‐node‐metastasis (TNM) stage in HCC patients. In addition, Kaplan‐Meier analysis revealed that patients with high DLEU1 expression had a significantly shorter overall survival compared to patients with low DLEU1 expression (Figure 1C). These results indicated that DLEU1 might be involved in HCC progression.

### 3.2 Knockdown of DLEU1 inhibits HCC cell proliferation, colony formation and cell cycle distribution

**Figure 2:**
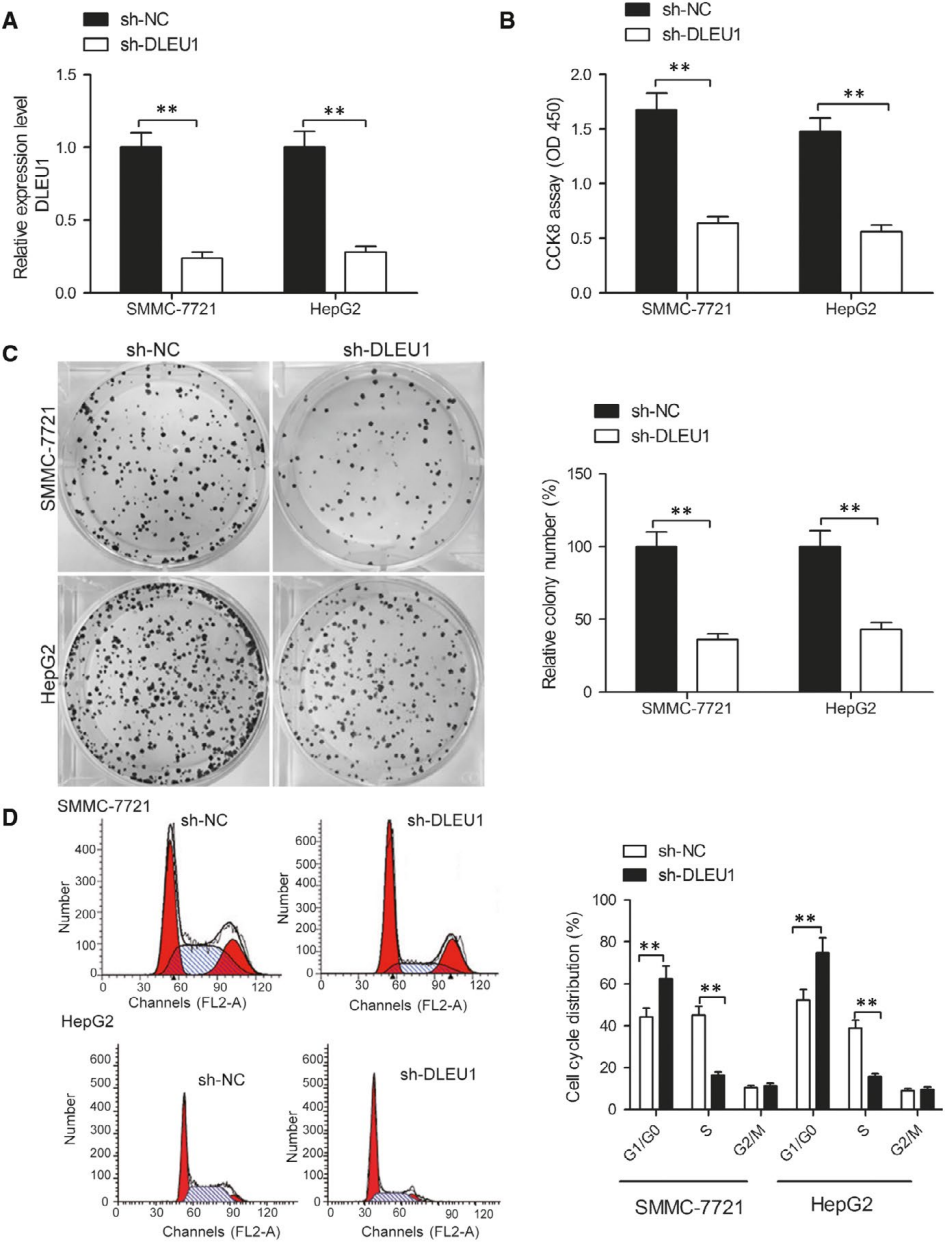
Knockdown of deleted in lymphocytic leukaemia 1 (DLEU1) inhibits hepatocellular carcinoma (HCC) cell proliferation, colony formation and cell cycle distribution. A, Depletion efficiency of SMMC‐7721 and HepG2 cells transfected with sh‐DLEU1 or sh‐NC was examined by quantitative real‐time polymerase chain reaction (qRT‐PCR). B, Cell Counting kit‐8 assay was used to evaluate the effect of DLEU1 on SMMC‐7721 and HepG2 cells proliferation. C, Colony formation assay was used to evaluate the effect of DLEU1 on SMMC‐7721 and HepG2 cells colony formation. D, Flow cytometry was used to evaluate the effect of DLEU1 on SMMC‐7721 and HepG2 cells cell cycle distribution. **P < 0.01

To evaluate the biological roles of DLEU1 on HCC progression, plasmid sh‐DLEU1 or sh‐NC were introduced into SMMC‐7721 or HepG2 cells, subsequently, cell proliferation, colony formation and cell arrest were detected. As shown in Figure 2A, sh‐DLEU1 produced a significant reduction in endogenous DLEU1 expression in SMMC‐7721 and HepG2 cells. After knockdown, both SMMC‐7721 and HepG2 cell proliferation were significantly suppressed in a CCK‐8 assay (Figure 2B). We similarly found that SNHG3 knockdown clearly decreased SMMC‐7721 and HepG2 cell colony formation (Figure 2C).Flow cytometry assay revealed that knockdown of DLEU1 lead to a significant reduction in the percentage of S phase and promotion in the percentage of G1/G0 phase in SMMC‐7721 and HepG2 cells compared to sh‐NC group (Figure 2D).

### 3.3 Knockdown of DLEU1 impairs HCC cell migration and invasion

**Figure 3:**
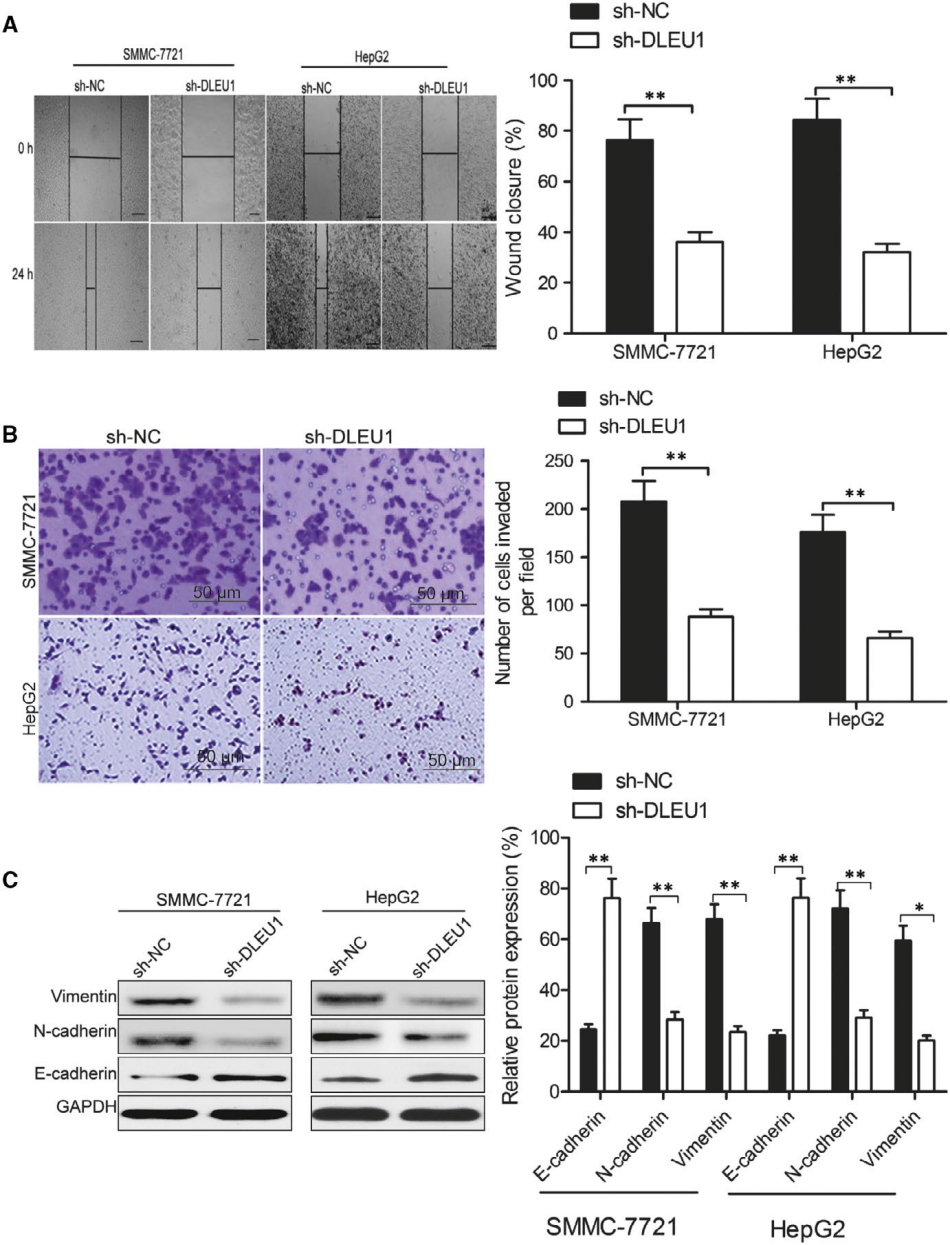
Knockdown of deleted in lymphocytic leukaemia 1 (DLEU1) inhibits hepatocellular carcinoma (HCC) cell migration and invasion. A, Wound healing assay was used to evaluate the effect of DLEU1 on SMMC‐7721 and HepG2 cells migration. B, Transwell invasion assay was used to evaluate the effect of DLEU1 on SMMC‐7721 and HepG2 cells invasion. C, Western blot analysis was applied to examine the effect of DLEU1 on epithelial to mesenchymal transition markers expression in SMMC‐7721 and HepG2 cells. *P < 0.05, **P < 0.01

We next used wound healing and invasion assays to explore how DLEU1 impacts HCC cell migration and invasion. The results showed that knockdown of DLEU1 could significantly suppress migration (Figure 3A) and invasion (Figure 3B) abilities in SMMC‐7721 and HepG2 cells. Furthermore, we explore the impacts of DLEU1 on epithelial‐mesenchymal transition (EMT) processes. The results of western blot demonstrated that knockdown of DLEU1 increased E‐cadherin expression, and decreased Vimentin and N‐cadherin expression in both SMMC‐7721 and HepG2 cells (Figure 3C).

### 3.4 DLEU1 was a target of miR‐133a in HCC cells

**Figure 4:**
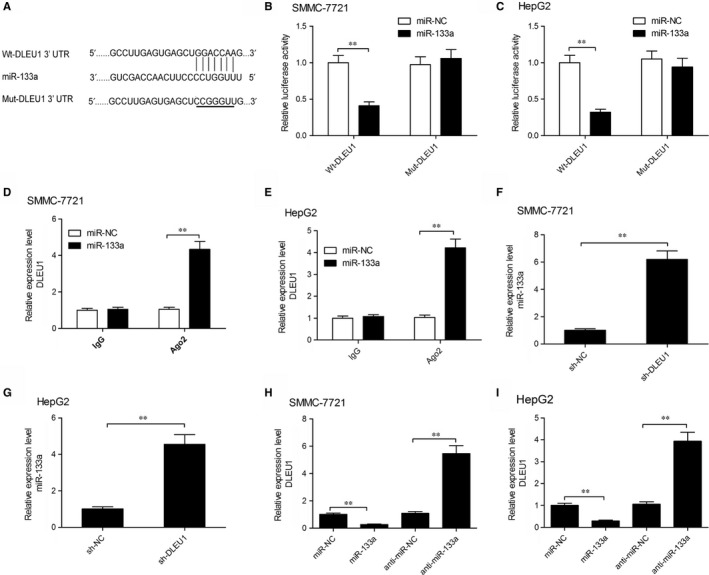
Deleted in lymphocytic leukaemia 1 (DLEU1) was a target of miR‐133a in hepatocellular carcinoma (HCC) cells. A, The predicted wild‐type or mutated miR‐133a binding sites in DLEU1. (B,C) Luciferase activity was determined in SMMC‐7721 and HepG2 cells after cotransfection with Wt‐DLEU1 or Mut/DLEU1 and miR‐133a mimic/ miRNA negative control (miR‐NC). (D,E) RNA immunoprecipitation assay was performed to evaluate the endogenous binding between DLEU1 and miR‐133a in SMMC‐7721 and HepG2 cells using specific antibody against Ago2, followed by measurement of RNA levels by quantitative real‐time polymerase chain reaction (qRT‐PCR). (F,G) Expression of miR‐133a was examined in SMMC‐7721 and HepG2 cells transfected with sh‐DLEU1 and sh‐NC by qRT‐PCR. (H,I) Expression of DLEU1 was examined in SMMC‐7721 and HepG2 cells transfected with miR‐133a mimic, miR‐NC, miR‐133a inhibitor (anti‐miR‐133a) and anti‐miR‐NC. **P < 0.01

Long non‐coding RNAs have been reported to function as a miRNA sponge in regulating the expression pattern and biological functions of miRNA.^5^ Thus, the potential target miRNA that may interact with DLEU1 was predicted by Starbase2.0. The result demonstrated that miR‐133a contained complementary binding sequences of DLEU1 (Figure 4A). To determine this prediction, dual luciferase reporter assay was performed. The result revealed that miR‐133a mimic significantly decreased the luciferase activity of Wt‐DLEU1, but not that of Mut‐DLEU1 in SMMC‐7721 (Figur4B) and HepG2 cells (Figur4C), indicating DLEU1 directly binds to miR‐133a in HCC cells. RNA immunoprecipitation assay displayed that both DLEU1 and miR‐133a tended to be enriched in Ago2‐containing beads compared with that of control IgG in both SMMC‐7721 (Figure 4D) and HepG2 cells (Figure 4E). Moreover, we found that silencing of DLEU1 lead to the up‐regulation of miR‐133a in both SMMC‐7721(Figure 4F) and HepG2 (Figure 4G) cells. In addition, miR‐133a overexpression obviously decreased DLEU1 expression, and underexpression of miR‐133a increased DLEU1 expression in both SMMC‐7721(Figure 4H) and HepG2 (Figure 4I) cells. Taken together, these data indicated that miR‐133a could directly bind to DLEU1 in HCC cells.

### 3.5 miR‐133a inhibitor rescued the inhibitory effect of HCC cells induced by DLEU1 depletion

**Figure 5:**
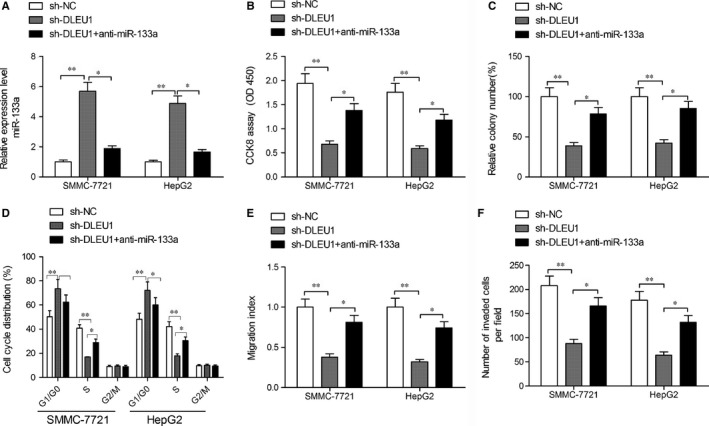
MiR‐133a inhibition rescued the inhibitory effect of hepatocellular carcinoma (HCC) cells induced by deleted in lymphocytic leukaemia 1 (DLEU1) depletion. A, Expression of miR‐133a was examined in SMMC‐7721 and HepG2 cell transfected with sh‐NC, sh‐DLEU1 and sh‐DLEU1 + anti‐miR‐133a by quantitative real‐time polymerase chain reaction (qRT‐PCR). (B‐F) Cell proliferation, colony formation, cycle, migration and invasion were determined in SMMC‐7721 and HepG2 cell transfected with sh‐NC, sh‐DLEU1 and sh‐DLEU1 + anti‐miR‐133a. *P < 0.05, **P < 0.01

To explore whether DLEU1 exerts biological functions through regulating miR‐133a, we performed rescue experiment by inhibiting miR‐133a expression in DLEU1 depletion‐SMMC‐7721 or HepG2 cells (Figure 5A). Moreover, we found that miR‐133a inhibition partially reversed the inhibitory effect on cell proliferation, colony formation, cycle arrest, migration and invasion caused by DLEU1 depletion in both SMMC‐7721 and HepG2 cells (Figur5B‐F). These findings indicated that DLEU1 promoted HCC development partially by regulating miR‐133a.

### 3.6 DLEU1 regulated IGF‐1R expression and PI3K/AKT signal pathway via inhibition of miR‐133a

**Figure 6:**
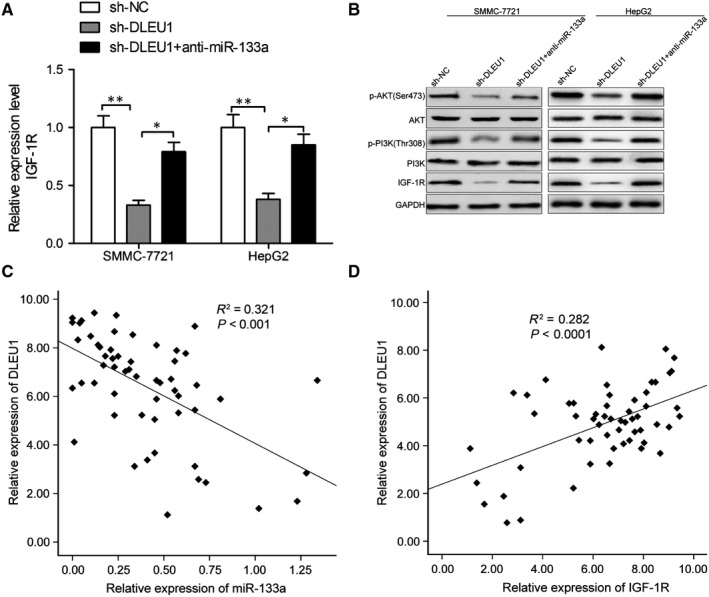
Deleted in lymphocytic leukaemia 1 (DLEU1) regulated IGF‐1R expression and PI3K/AKT signal pathway via regulation of miR‐133a. A, *IGF1R* mRNA expression was examined in SMMC‐7721 and HepG2 cells transfected with sh‐NC, sh‐DLEU1 and sh‐DLEU1 + anti‐miR‐133a by quantitative real‐time polymerase chain reaction (qRT‐PCR). B, IGF‐1R, PI3K, p‐PI3K, AKT and anti‐phospho‐Akt (p‐AKT) proteins were examined in SMMC‐7721 and HepG2 cells transfected with sh‐NC, sh‐DLEU1 and sh‐DLEU1 + anti‐miR‐133a by western blot. C, Pearson's correlation analysis between DLEU1 and miR‐133a expression in hepatocellular carcinoma tissues (n = 56). D, Pearson's correlation analysis between DLEU1 and *IGF‐1R* mRNA expression in HCC tissues (n = 56). *P < 0.05, **P < 0.01

Insulin‐like growth factor 1 receptor, a known oncogene, was identified to serve as a downstream of miR‐133a in HCC in our previous study.^18^ Therefore, we wonder whether DLEU1 regulated IGF‐1R via regulating miR‐133a in HCC cells. sh‐NC, sh‐DLEU1 and sh‐DLEU1 + miR‐133a inhibitor were separately transfected into SMMC‐7721 or HepG2 cells, then IGF‐1R expression on mRNA and protein levels was determined by qRT‐PCR and western blot analyses respectively. Our results demonstrated that knockdown of DLEU1 led to a significant decrease of IGF‐1R mRNA (Figure 6A) and protein expression (Figure 6B) in both SMMC‐7 and HepG2 cells, while miR‐133a inhibitor partially reversed this trend. It was well known that IGF‐1R could activate PI3K/AKT signalling pathways in HCC cells.^18^, ^19^ Here, we investigated whether DLEU1 affect activation of PI3K/ AKT pathway. We found that knockdown of DLEU1 significantly inhibited activation of PI3K/ AKT pathway in SMMC‐7721 and HepG2 cells (Figure 6B), while miR‐133a inhibitor reversed the trends. We further investigated the correlation between the DLEU1, miR‐133a and IGF‐1R in HCC clinical samples. We found that DLEU1 expression was negatively correlated with miR‐133a expression (Figure 6C), while its expression was positively correlated with IGF‐1R expression in HCC tissues (Figure 6D). Collectively, these data suggest that DLEU1 modulated IGF‐1R and PI3K/AKT pathway via regulation of miR‐133a in HCC.

### 3.7 Knockdown of DLEU1 suppressed tumour growth in vivo

**Figure 7:**
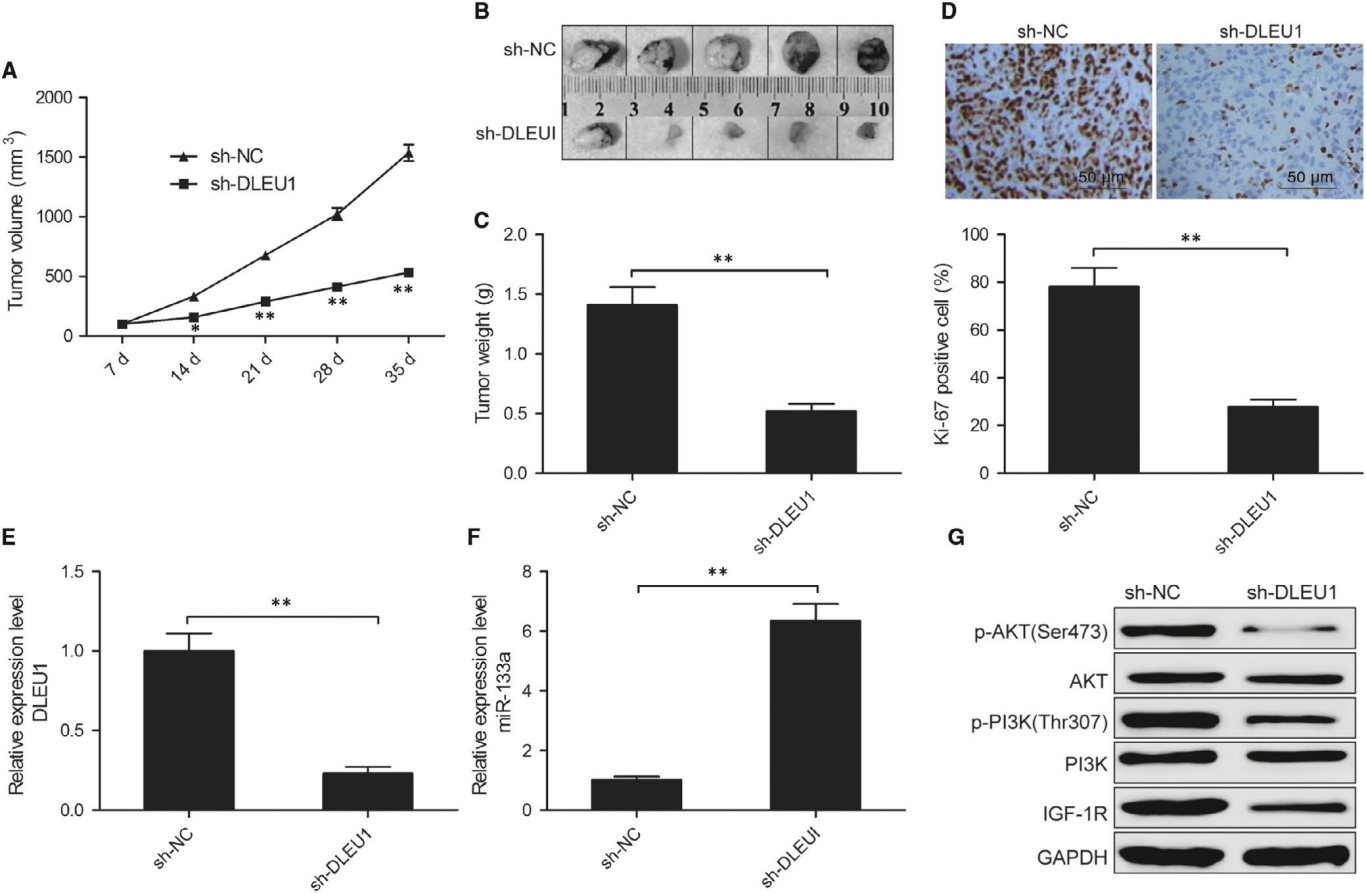
Knockdown of deleted in lymphocytic leukaemia 1 (DLEU1) inhibits tumour growth in vivo. A, Tumour growth curve. B, Representative images of xenograft tumours. C, Tumour weight. D, Representative images of immunohistochemistry staining patterns for Ki‐67 in xenograft tumour.(E,F) DLEU1 and miR‐133a expression were examined in xenograft tumour by quantitative real‐time polymerase chain reaction (qRT‐PCR). G, IGF‐1R, PI3K, p‐PI3K, AKT and anti‐phospho‐Akt (p‐AKT) proteins expression were examined in xenograft tumour by western blot. *P < 0.05, **P < 0.01

To validate the functional relevance of DLEU1 in vivo, a xenograft tumour model of HCC was established by subcutaneously injecting SMMC‐7721 cells stably transfected with sh‐DLEU1 or sh‐NC. As presented in Figure 7A, DLEU1 knockdown significantly inhibited tumour growth in nude mice compared to sh‐NC group. Also, knockdown of DLEU1 evidently impaired tumour size (Figur7B) and weight (Figure 7C) in contrast with sh‐NC group. Immunohistochemistry (IHC) was performed to analyse the Ki‐67 (a proliferation index) expression in xenograft tumours. As shown in Figure 7D, Ki‐67‐positive cells were significantly decreased in sh‐DLEU1 group compared with sh‐NC group. We also examined the expression of DLEU1 and miR‐133a in xenograft tumours. Our results showed that DLEU1 expression was obviously down‐regulated (Figure 7E), while miR‐133a expression was up‐regulated in sh‐DLEU1 group compared with sh‐NC group (Figure 7F). In addition, we also investigated the effect of DLEU1 on IGF‐1R expression and its downstream PI3K/AKT pathway in xenograft tumour. We found that silencing of DLEU1 significantly decreased IGF‐1R expression and its downstream PI3K/AKT pathway (Figure 7G). These results support the growth‐promoting effect of DLEU1 in HCC in vivo.

## 4 DISCUSSION

Accumulating evidence indicated that lncRNAs had crucial roles in occurrence and development of HCC, which is attracting more and more attention to find valuable diagnostic and prognostic biomarkers for HCC.^20^, ^21^ In the present study, we found that DLEU1 was significantly up‐regulated in HCC tissues and cell lines, and up‐regulated DLEU1 was closely associated with TNM stage, vascular metastasis and poor overall survival ratio. In addition, we found that knockdown of DLEU1 exerted suppressive effects on HCC progression by regulating miR‐133a/IGF‐1R axis. To the best our knowledge, this study first showed a crucial role for DLEU1 in HCC tumourigenesis, suggesting that DLEU1 might be a potential therapeutic target for HCC.

Deleted in lymphocytic leukaemia 1, as an oncogenic lncRNA, has been shown to be involved in the progression of multiple cancers by different mechanisms of action.^11^, ^12^, ^13^, ^14^, ^15^, ^16^. For example, Liu et al reported that DLEU1 promoted colorectal cancer growth by regulating SMARCA1/KPNA3 axis.^13^ Shao et al demonstrated that DLEU1 drove the development of endometrial cancer by interacting with miR‐490 to regulate SP1 expression and PI3K/AKT/GSK‐3β pathway.^16^ Wang et al reported that DLEU1 promoted ovarian cancer tumourigenesis and development by sponging miR‐490‐3p and altering CDK1 expression.^15^ However, the effects of DLEU1 on HCC, and its underlying molecular mechanisms remain unclear. We firstly examined the expression of DLEU1 in HCC tissues and adjacent normal tissues by qRT‐PCR, and found that DLEU1 expression in HCC tissues was significantly higher than that in adjacent normal tissues. In addition, up‐regulation DLEU1 was positively correlated with advanced TNM stage, vascular metastasis and poor overall survival ratio. Loss‐of‐function assay was performed by the knockdown of DLEU1 in HCC cells to investigate the biological functions of DLEU1. Knockdown of DLEU1 could impair HCC cell proliferation, colony formation, migration and invasion. The nude mice xenograft assays further revealed that knockdown of DLEU1 suppressed tumour growth in vivo. These results suggested that DLEU1 functioned as an oncogene that promotes HCC progression.

Accumulating evidence demonstrated that lncRNAs could function as endogenous miRNA sponges or competing endogenous RNA (ceRNAs) by binding to miRNAs and regulating their function.^22^, ^23^ Therefore, we applied the Starbase2.0 software to identify the miRNAs that could bind to complementary sequences in DLUE1. We found that miR‐133a shares the complementary binding sites at DLEU1 3′‐UTR, which was confirmed by the luciferase assay, RIP assay and qRT‐PCR assay. Our published study showed that miR‐133a expression was down‐regulated in HCC, and functioned as tumour suppressor in HCC progression.^18^ Moreover, the present study demonstrated that miR‐133a inhibitor partially reversed the inhibitory effect caused by DLEU1 depletion. These results suggested that DLEU1 exerts tumour‐promoting function in HCC via partially sponging miR‐133a.

It has been shown that LncRNAs can affect the expression and biological functions of miRNA targets.^24^, ^25^ miR‐133a was reported to exert tumour suppressor role in HCC by regulating IGF‐1R^18^ Growing evidence has supported the role of IGF‐1R in promoting carcinogenesis and act as oncogene in multiple cancers including HCC.^26^ In addition, abnormal expression of IGF‐1R could regulate multiple downstream signal pathways including PI3K/AKT pathway.^27^, ^28^ Here, we found that knockdown of DLEU1 significantly decreased IGF‐1R expression and inhibited the activation of PI3K/AKT pathway in SMMC‐7721 and HepG2 cells, while miR‐133a inhibitor reversed this trends. Moreover, we found that DLEU1 expression was positively correlated with IGF‐1R expression in HCC tissues. Interestingly, we found that knockdown of DLEU1 significantly inhibited DLEU1 expression, increased miR‐133a expression, and inhibited IGF‐1R expression and its downstream PI3K/AKT pathway in xenograft tumours. These data suggest that DLEU1 modulated IGF‐1R and PI3K/AKT pathway via regulating miR‐133a in HCC.

In conclusion, the present study demonstrated that DLEU1 was highly expressed in HCC tissues and its up‐regulation was closely associated with TNM stage, vascular metastasis and poor overall survival ratio. DLEU1 could serve as an oncogenic lncRNA that promoted HCC tumourigenesis via acting as a ceRNA to regulate the expression of IGF‐1R and its downstream PI3K/AKT signal pathway through directly sponging for miR‐133a. These findings implied that DLEU1 might be a potential therapeutic target for HCC.

## CONFLICT OF INTEREST

The authors declare that they have no conflict of interest.

## AUTHOR'S CONTRIBUTIONS

Yahui Liu was responsible for the conception and design of the study. Wei Zhang performed the experiments. Kai Liu analysed and interpreted the data. Songyang Liu wrote and revised the manuscript. All the authors read and approved the final manuscript.

## DATA AVAILABILITY STATEMENT

All data generated or analysed during the present study are included in this published article.
